# Unmet mental health care need 10–11 years after the 9/11 terrorist attacks: 2011–2012 results from the World Trade Center Health Registry

**DOI:** 10.1186/1471-2458-14-491

**Published:** 2014-05-22

**Authors:** Sharon J Ghuman, Robert M Brackbill, Steven D Stellman, Mark R Farfel, James E Cone

**Affiliations:** 1 New York City Department of Health and Mental Hygiene, 42-09 28th Street, Long Island City, NY 11101, USA; 2 Department of Epidemiology, Mailman School of Public Health, Columbia University, New York, NY, USA

**Keywords:** Unmet mental health care need, World Trade Center disaster, September 11, 2001, 9/11, Barriers to mental health care, Posttraumatic stress disorder (PTSD), Depression, Comorbid PTSD and depression, Functional impairment

## Abstract

**Background:**

There is little current information about the unmet mental health care need (UMHCN) and reasons for it among those exposed to the World Trade Center (WTC) terrorist attacks. The purpose of this study was to assess the level of UMHCN among symptomatic individuals enrolled in the WTC Health Registry (WTCHR) in 2011–2012, and to analyze the relationship between UMHCN due to attitudinal, cost, and access factors and mental health symptom severity, mental health care utilization, health insurance availability, and social support.

**Methods:**

The WTCHR is a prospective cohort study of individuals with reported exposure to the 2001 WTC attacks. This study used data from 9,803 adults who completed the 2003–2004 (Wave 1) and 2011–2012 (Wave 3) surveys and had posttraumatic stress disorder (PTSD) or depression in 2011–2012. We estimated logistic regression models relating perceived attitudinal, cost and access barriers to symptom severity, health care utilization, a lack of health insurance, and social support after adjusting for sociodemographic characteristics.

**Results:**

Slightly more than one-third (34.2%) of study participants reported an UMHCN. Symptom severity was a strong predictor of UMHCN due to attitudinal and perceived cost and access reasons. Attitudinal UMHCN was common among those not using mental health services, particularly those with relatively severe mental health symptoms. Cost-related UMHCN was significantly associated with a lack of health insurance but not service usage. Access-related barriers were significantly more common among those who did not use any mental health services. A higher level of social support served as an important buffer against cost and access UMHCN.

**Conclusions:**

A significant proportion of individuals exposed to the WTC attacks with depression or PTSD 10 years later reported an UMHCN, and individuals with more severe and disabling conditions, those who lacked health insurance, and those with low levels of social support were particularly vulnerable.

## Background

The mental health consequences of terrorism are the subject of a sizable literature, but only a few studies are devoted to the unmet mental health care need (UMHCN) of those affected [1-3]. To our knowledge, there is no current information on the UMHCN of those exposed to the September 11, 2001 World Trade Center (WTC) attacks. Studies conducted in New York City (NYC) within 2 years of the disaster showed that people with mental health symptoms delayed or failed to seek treatment due to financial and access problems and a reluctance to seek care [4,5]. A renewed interest in the UMHCN of exposed persons has arisen due to the recent release of public funds for monitoring and treatment of 9/11-related health conditions, which includes the provision of mental health services at no out of pocket cost for those who are eligible [6].

The prevalence of probable posttraumatic stress disorder (PTSD) after a disaster varies by the type and severity of exposure, individual risk factors, and the study methodology [7]. In previous disaster studies that used random or systematic samples, the prevalence of PTSD among adults varied from less than 5% to as high as 40% among highly exposed survivors of the 1989 Newcastle earthquake [8]. Numerous studies have indicated a substantial burden of disease due to mental health problems among persons with reported exposure to 9/11 [9-12]. In the WTC Health Registry (WTCHR) cohort, 19% of individuals who had no prior history of PTSD exhibited PTSD symptoms 5 to 6 years after the disaster [13]. An application of this rate to the total persons exposed to 9/11 as defined by the Registry indicates an estimated 61,000 adults with probable PTSD [13,14]. The National Comorbidity Survey Replication indicated a median lag of 12 years between PTSD onset and initial treatment contact [15]. Therefore, the decision to seek mental health care among those exposed to 9/11 is likely to be protracted with many symptomatic individuals only recently considering treatment. The delay in both onset of mental health symptoms and initiation of treatment indicates the need for a current assessment of the UMHCN of those exposed to the 9/11 attacks.

The importance of assessing UMHCN now among 9/11-affected individuals arises in the context of several trends. Contact with health care professionals for mental health issues has increased as more people with mental illnesses are covered by insurance [16]. Nevertheless, nearly one in three (32%) persons with mental health disorders such as major depression report UMHCN, and symptomatic individuals increasingly cite cost as a barrier to obtaining care [17,18]. In addition, attitudes indicating low treatment acceptability have lessened only modestly over time, and among those with symptoms, are associated with not seeking and failing to complete treatment [19-22]. Findings from the WTCHR showed that 5 to 6 years after 9/11, 20% of individuals with a diagnosed mental health condition reported UMHCN [1]. UMHCN was furthermore found to be associated with lower quality of life as measured by poor mental health days and low levels of social support.

There are important gaps in the current knowledge of UMHCN among those exposed to 9/11. In this study we consider three major types of UMHCN related to attitudinal, access and cost barriers. Information is lacking on the prevalence of each type of UMHCN due to attitudinal (e.g. being afraid to ask for help), cost, or access (e.g. an inability to locate a provider) factors, and on the relationship between each type of UMHCN and the severity of illness. We used 2011–2012 data on individuals with probable PTSD or depression from the WTCHR to examine the relationship between UMHCN and sociodemographic, health care (use of mental health services, lack of health insurance), and social support variables. A second aim was to investigate the UMHCN of those with a greater burden of illness, and the relationship between type of UMHCN and symptom severity as measured by the presence of comorbid PTSD and depression and functional impairment. Finally, we analyzed the association of attitudinal, cost, and access UMHCN with health care utilization, symptom severity, the availability of health insurance, and social support.

## Methods

### Study population

The WTCHR is a cohort study designed to monitor the physical and mental health status and health care need of a diverse population with reported exposure to the 9/11 attacks in NYC. A detailed description of study methods is available elsewhere [14]. The sample was identified using lists provided by employers and government agencies (30% “list-identified”) or was recruited through outreach campaigns (70% “self-identified”). In 2003–2004 (Wave 1), a total of 71,434 individuals completed an interview via telephone (95%) or in person (5%). Registry coverage of the total eligible population of persons potentially physically exposed to WTC dust and debris is an estimated 17%, varying from 34% among rescue and recovery workers, 26% of residents south of Canal Street, and 12% of building occupants, passersby, and people in transit south of Chambers Street on September 11, 2001 [14]. In 2006–2007, the Wave 2 survey was completed by 46,602 individuals who also had a complete interview upon enrollment in 2003–2004 (Wave 1) [23]. The Wave 3 survey was completed in 2011–2012 by 43,134 individuals [24]. The Centers for Disease Control and Prevention (CDC) and the New York City Department of Health and Mental Hygiene (DOHMH) institutional review boards approved the study protocol.

This analysis focused on adults who completed the 2011–2012 (Wave 3) surveys and had symptoms of probable PTSD or depression in Wave 3. The prevalence of PTSD or depression in the adult population who completed Wave 3 was 23%. We excluded the small number (n = 14) of individuals who were staff or students in schools south of Canal Street and were not in any other eligibility group. The final study sample was 9,803 individuals.

### Study variables

The Wave 1 interview provided data on sociodemographic characteristics: eligibility group (rescue and recovery workers, residents, and building occupants or those in transit), gender, race/ethnicity, education level, and household income in 2002. UMHCN, mental health, health care, and social support variables are from the Wave 3 survey and thus represent the most recent assessment of mental health symptoms and UMHCN of the Registry cohort.

Perceived UMHCN is signified by a response of “yes” to the following item: “During the last 12 months, was there ever a time when you needed mental health care or counseling, but didn’t receive it?” Persons who answered “yes” were offered a list of nine reasons for UMHCN (Table 1), which we classified into one of three categories based on previous literature and correlations between the nine reasons for UMHCN [22]. An individual who selected at least one of the four reasons listed under the attitudinal category was classified as having this type of UMHCN. Cost UMHCN was defined as selecting at least one of two cost-related reasons for UMHCN (i.e. “couldn’t afford to pay” or “no insurance or not covered by insurance”) and access UMHCN was defined as selecting at least one of the three reasons for this type of need shown.

**Table 1:** Reasons for UMHCN by type, World Trade Center Health Registry, 2011–2012

***UMHCN Type***	***Reason***
***Attitudinal***	Preferred to manage myself, didn’t think anything could help, afraid to ask for help or of what others would think, didn’t get around to it or didn’t bother
***Cost***	Couldn’t afford to pay, no insurance or not covered by insurance
***Access***	Did not know where to go or what kind of doctor to go to for care, problems with transportation, scheduling, childcare, or other family responsibilities, unable to find a provider who could diagnose or treat condition

UMHCN, unmet mental health care need.

Probable PTSD was assessed with the stressor-specific PTSD Checklist-Civilian version (PCL). The PCL consists of 17 items based on the *Diagnostic and Statistical Manual of Mental Disorders (Fourth Edition)* criteria that are linked to exposure to the events of 9/11 [25]. Each item refers to symptoms during the past 30 days and is scored on a scale from 1 to 5. Consistent with prior published Registry analyses, a score of 44 or greater on the PCL indicated probable PTSD [26,27]. Depression was assessed with the 8-item Patient Health Questionnaire (PHQ). Each item refers to symptoms in the past 2 weeks and is scored from 0 to 4, with a score above 10 indicating probable moderate to severe depression [28]. Comorbidity was defined as the co-occurrence of probable PTSD and depression.

### Symptom severity

Symptom severity was derived from functional impairment levels and the presence of a comorbid mental health condition and comprises a four category measure ranging from “low” to “very high” (Table 2). Functional impairment is assessed after the PCL with the following questionnaire item: “Thinking about the previous questions, how difficult have these problems made it for you to do your work, take care of things at home, or get along with other people?” Individuals are also asked whether their problems lasted for longer than 1 month continuously in the previous year. We classified a response of either “no difficulty” or any problem lasting for less than a month as “none or low” functional impairment. A response of “somewhat difficult” was categorized as “medium” impairment and a response of “very or extremely difficult” as “high” impairment.

**Table 2:** Symptom severity level by functional impairment and presence or absence of comorbid mental health condition

**Comorbid PTSD and depression**	**Levels of functional impairment**^**a**^		
	**None or low**	**Medium**	**High**
Yes	Medium severity	High severity	Very high severity
No	Low severity	Medium severity	High severity

^a^See text for definition.

### Health care variables

Use of mental health services was defined as having seen a doctor or health professional or taking any prescription medicine in the preceding year for depression, PTSD, anxiety disorder other than PTSD or nerves, emotions, and other mental health problems. Individuals were classified according to whether they lacked health insurance at any point in the preceding year.

### Social support

We considered the main dimensions of social support from the literature on sociology and psychology including “perceived” support, or one’s assessment of the emotional support received from social networks and “received” support, which refers to the instrumental or informational assistance provided by one’s network [29]. In a previous study of social support and UMHCN, different elements of perceived and received support such as “tangible” support (e.g. assistance with meal preparation), “affection”, “positive social interaction” (e.g. someone to have a good time with), and “emotional support” (e.g. someone to understand one’s problems) were considered separately [22]. There are five items in the Registry questionnaire that pertain to perceived or received support including: how often someone is available to take the respondent to the doctor, have a good time with them, hug them, prepare meals if they are unable to do it themselves, and understand their problems. We calculated Cronbach’s alpha for these five items. Cronbach’s alpha is a common estimate of the internal reliability of a psychometric test and ranges from 0 to 1 [30]. The alpha estimate for these items in our sample was 0.88, indicating that they have a high degree of internal consistency. Accordingly, an index of social support was created by summing the five items; the index ranges from 0 to 20 and for the analysis, scores were classified into quartiles.

### Statistical analysis

We calculated the prevalence of UMHCN by sociodemographic, mental health, health care, and social support variables. The statistical significance of the association of each variable with UMHCN was tested with a chi-square statistic and is indicated by a p-value that is less than .01 or .05. We then calculated the prevalence of attitudinal, cost, and access UMHCN for each level of symptom severity and further stratified the prevalences by use and non-use of mental health services in the preceding year. Multivariate logistic models were used to show the association between the three types of UMHCN and symptom severity, health care variables (use of mental health services and lack of health insurance), and social support after controlling for sociodemographic characteristics. We tested for interaction terms between symptom severity and the use of mental health services and retained statistically significant interaction terms in the final models.

All analyses were conducted using SAS version 9.2.

## Results

### Sample characteristics and UMHCN

Among the 9,803 individuals with PTSD or depression in the Wave 3 Registry survey, 47.7% were rescue and recovery workers, 39.9% were building occupants or in transit on the morning of 9/11, and 12.4% were residents south of Canal Street (Table 3). The sample was 58.2% male, 61.8% white, 43.5% college graduates, and 62.6% had a household income of at least $50,000 in 2002. One-half of study individuals had comorbid PTSD and depression, and about two-thirds were functionally impaired for longer than 1 month in the past year. A very high level of symptom severity was observed in 20.5% of the study sample. One-half (49.8%) had used any mental health services and 11.9% lacked health insurance at any point in the preceding year.

**Table 3:** **UMHCN characteristics of respondents with PTSD or depression, World Trade Center Health Registry, 2011** - **2012**

	**N**	**%**	**UMHCN (%)**
Total	9,803	100	34.2
***Socio-demographic variables***			
*Eligibility group*			
Rescue and recovery	4,675	47.7	32.6*
Residents	1,214	12.4	38.1
Occupants or in transit	3,914	39.9	34.9
*Gender*			
Male	5,706	58.2	31.3*
Female	4,097	41.8	38.2
*Age (on Sept 11, 2001)*			
18-34	2,304	23.5	43.2*
35-44	3,586	36.6	35.2
45-54	2,889	29.5	28.8
55+	1,024	10.5	25.2
*Race*			
White (non-hispanic)	6,055	61.8	34.2†
Black (non-hispanic)	1,121	11.4	31.3
Hispanic or latino	1,664	17.0	35.8
Asian	507	5.2	32.3
Multiracial or other	456	4.7	38.1
*Education*			
Up to high school graduate	2,739	28.2	30.3*
Some college or technical school	2,746	28.3	33.2
College graduate	4,219	43.5	37.2
*Household income in 2002 ($000’s)*			
Less than 25	1,214	13.5	43.2*
25 to 50	2,160	24.0	37.2
50 to 75	2,037	22.6	31.2
75 to 100	1,601	17.8	30.3
100+	2,002	22.2	32.0
***Mental health variables***			
*Comorbid PTSD and depression*			
Yes	4,886	49.8	41.7*
No	4,917	50.2	26.8
*Level of functional difficulty*^*a*^			
None or low	3,269	33.9	21.7*
Medium	3,968	41.1	36.5
High	2,421	25.1	47.0
*Symptom severity*^*a*^			
Low	2,269	23.5	18.8*
Medium	3,108	32.2	31.1
High	2,306	23.9	41.0
Very high	1,975	20.5	48.6
***Health care variables***			
*Used any mental health services in past year*			
Yes	4,780	49.8	34.6
No	4,820	50.2	33.8
*Lacked insurance at any point in past year*			
Yes	1,132	11.9	59.6*
No	8,364	88.1	30.3
***Social support index***			
0 - 6	2,402	25.3	45.1*
7 - 11	2,528	26.6	36.6
12 - 15	2,263	23.9	30.8
16 - 20	2,296	24.2	22.7

UMHCN, unmet mental health care need.

Association with UMHCN is statistically significant at †p < .05 or *p < .01.

^a^See text for definition.

UMHCN was reported by slightly over one-third (34.2%) of the study sample. It was higher among women (38.2%) compared with men (31.3%), and was associated with younger age (i.e., 43.2% of 18–34 year olds reported UMHCN as compared to 25.2% of those 55 and over), higher education level (i.e., a college education), and lower household income in 2002. All of these associations are statistically significant with a p-value < .05. The severity of mental health symptoms, health care, and social support variables had similarly strong associations with UMHCN. The prevalence of UMHCN was 18.8% among those with low severity symptoms as compared to 31.1%, 41.0%, and 48.6% among those with medium, high, and very high severity symptoms. UMHCN was common among persons who lacked insurance in the preceding year (59.6%) as compared to 30.3% of those who did not lack insurance. Slightly over 45% of people with the lowest level of social support reported UMHCN as compared to 22.7% of those with the highest level of support.

### Association of UMHCN type with symptom severity

The percentage of persons who reported using any mental health services in the preceding year increased with symptom severity, from 34.6% of those with the lowest level of symptoms to 71.1% of those with the most intense symptom level (Table 4). Without exception, reported UMHCN increased monotonically from low to high symptom severity within mental health service use or non-use groups. For attitudinal UMHCN, the positive relationship with symptom severity was more pronounced among service non-users as compared to those who had used services in the preceding year, rising from 11.7% among those with low severity symptoms to 45.1% of those with the most severe symptoms.

**Table 4:** **UMHCN type by mental health service use and symptom severity, World Trade Center Health Registry, 2011** - **2012**

		**Symptom severity level (%)**			
		**Low**	**Medium**	**High**	**Very high**
		**(N = 2,185)**^**a**^	**(N = 3,008)**^**a**^	**(N = 2,214)**^**a**^	**(N = 1,908)**^**a**^
*UMHCN type*	Used mental health services in past year^b^	34.6	44.7	53.0	71.1
Attitudinal	Yes	14.3	18.1	21.1	22.7
	No	11.7	25.9	32.9	45.1
Cost	Yes	8.6	12.3	16.3	23.4
	No	5.0	11.9	18.4	27.4
Access	Yes	6.2	9.5	11.5	17.7
	No	5.3	9.7	16.0	24.5

UMHCN, unmet mental health care need.

^a^Individuals with complete data on all the variables shown in the table.

^b^Saw doctor or health professional or took any prescription medicine in the preceding year for depression, PTSD, anxiety disorder other than PTSD or nerves, emotions, and other mental health problem.

### Adjusted odds ratios for UMHCN by type

Table 5 shows the adjusted odds ratios (AORs) for the association of each type of UMHCN with symptom severity, health care variables, and social support after adjusting for sociodemographic characteristics. After accounting for missing values, the total sample size available for analysis in the multivariate models was 8,142. An interaction between symptom severity and the use of mental health services was only statistically significant (p-value <.05) for attitudinal UMHCN and was retained in the multivariate model for this type of need. Table 5 displays this interaction as the association of attitudinal UMHCN with all the possible combinations of symptom severity and the use of mental health services.

**Table 5:** **Adjusted odds ratios for association of UMHCN type with symptom severity, health care variables and social support, World Trade Center Health Registry 2011–2012 (N = 8,142)**^**a**^

	**Attitudinal UMHCN**_**b**_		**Cost UMHCN**		**Access UMHCN**	
	***OR***	***95% CI***	***OR***	***95% CI***	***OR***	***95% CI***
*Symptom severity*						
Low	b		Ref		Ref	
Medium	b		2.0	(1.6, 2.5)	1.9	(1.5, 2.5)
High	b		2.7	(2.2, 3.5)	2.6	(2.0, 3.3)
Very high	b		4.2	(3.3, 5.4)	4.1	(3.2, 5.2)
*Used any mental health services in past year*						
Yes	b		Ref		Ref	
No	b		1.0	(.9, 1.1)	1.3	(1.1, 1.5)
*Lacked insurance at any point in past year*						
Yes	1.1	(.9, 1.3)	8.2	(7.0, 9.7)	1.4	(1.1, 1.7)
No	Ref		Ref		Ref	
*Social support index*						
0 to 6	1.7	(1.4, 2.0)	2.9	(2.3, 3.6)	2.6	(2.1, 3.3)
7 to 11	1.5	(1.2, 1.7)	1.9	(1.5, 2.4)	1.8	(1.5, 2.3)
12 to 15	1.3	(1.1, 1.5)	1.6	(1.2, 2.0)	1.4	(1.1, 1.8)
16 to 20	Ref		Ref		Ref	
*Association of symptom severity by mental health service use*						
Low severity, not using services	Ref					
Medium severity, not using services	2.7	(2.2, 3.3)				
High severity, not using services	3.6	(2.9, 4.5)				
Very high severity, not using services	5.8	(4.5, 7.5)				
Low severity, using services	1.2	(.9, 1.6)				
Medium severity, using services	1.6	(1.2, 2.0)				
High severity, using services	2.0	(1.5, 2.5)				
Very high severity, using services	2.1	(1.7, 2.6)				

UMHCN, unmet mental health care need. ^a^Adjusted for eligibility group, gender, age on September 11, 2011, race, education, and household income in 2002. ^b^Coefficients are shown below.

The analysis confirmed a strong association of symptom severity with attitudinal UMHCN. Among individuals who had not used any mental health services in the preceding year, those with medium, high, and very high severity symptoms were 2.7 (95% CI = 2.2, 3.3), 3.6 (95% CI = 2.9, 4.5), and 5.8 (95% CI = 4.5, 7.5) times more likely to perceive an attitudinal UMHCN than those who had low severity symptoms. Persons with medium, high, and very high severity symptoms who were using services were also 1.6 (95% CI = 1.2, 2.0), 2.0 (95% CI = 1.5, 2.5), and 2.1 (95% CI = 1.7, 2.6) times more likely to report an attitudinal UMHCN than the low severity group that had not used any mental health services.

Cost and access UMHCN had strong dose–response associations with symptom severity, and the magnitude of the relationship was similar for both types of need. The AORs for cost UMHCN were 2.0 (95% CI = 1.6, 2.5) for medium severity, 2.7 for high severity (95% CI = 2.2, 3.5), and 4.2 for very high severity symptom level (95% CI = 3.3, 5.4). Cost UMHCN was 8.2 times higher among individuals who lacked health insurance at any time in the preceding year (95% CI = 7.0, 9.7). In addition, access UMHCN was 1.4 times more likely among those who lacked insurance (95% CI = 1.1, 1.7) and 1.3 times higher among service non-users (95% CI = 1.1, 1.5).

Social support was strongly associated with UMHCN and the magnitude of the relationship of cost and access UMHCN with social support was similar. The AORs for cost UMHCN were 2.9 for a score of 0 to 6 on the social support index (95% CI = 2.3, 3.6), 1.9 for values of 7 to 11 (95% CI = 1.5, 2.4), and 1.6 for a score of 12 to 15 (95% CI = 1.2, 2.0). Attitudinal UMHCN was also associated with lower levels of social support, although the strength of the association was weaker as compared to cost and access UMHCN.

## Discussion

Individuals in the WTCHR cohort with PTSD or depression at the time of the 2011–2012 survey continue to display a significant burden of UMHCN and poor quality of life due to mental health conditions. Recent findings from the Wave 3 (2011–2012) WTCHR survey showed that slightly more than one in three individuals with PTSD or depression reported an UMHCN, a level similar to that reported in previous nationally representative samples of individuals with mental health symptoms [17,31]. Two-thirds of study participants reported poor quality of life in terms of functional impairment that lasted for longer than a month in the preceding year. The severity of mental health symptoms as measured by functional impairment and comorbidity was strongly associated with perception of an attitudinal, cost, and access-related UMHCN. The association of UMHCN and symptom severity remained strong after controlling for a wide range of sociodemographic variables, mental health care utilization, the availability of health insurance, and social support.

We found that perceived attitudinal barriers to seeking care were common among individuals with relatively severe mental health symptoms who had not used services. Previous studies have also shown that attitudinal barriers, such as wanting to handle a problem on their own and ambivalence about the effectiveness or quality of mental health care, are frequently cited as reasons for not initiating and discontinuing treatment by individuals with severe mental health conditions [15,20]. Cost-related barriers were common among service users and non-users and were associated with a lack of health insurance. Access problems, such as not knowing where to go for care or an inability to find an appropriate provider, were more common among mental health service non-users, suggesting that they serve as important deterrents to seeking care. Consistent with previous studies, we found that social support serves as a protective factor against cost and access UMHCN in particular, which likely reflects its role in reducing the informational, logistical, and affordability barriers involved in treatment initiation and continuation [21,32].

UMHCN as perceived by individuals is but one way to measure the complex concept of need for mental health care [33]. Reliance on direct individual assessment of need avoids the assumption that the presence of a mental health symptom or the non-use of mental health services indicates unmet need for treatment [22]. A limitation of this measure of unmet need is that individual perception is subjective and people may not report a need if, for example, they are uninformed about treatment options, fear disclosing their condition or lack the resources to seek care [31]. However, the strong association of perceived UMHCN with the severity of mental health symptoms shown in this study and in previous literature indicates that this measure still conveys important information about vulnerability and in turn, the need for care [17,21].

These findings show that there is substantial UMHCN among individuals with mental health symptoms who were exposed to the WTC attack despite the widespread availability of treatment for 9/11-related mental health conditions. This suggests that expanded outreach is necessary to reach these individuals and inform them about treatment options and to encourage them to seek care. For example, the access barriers we report in this study (e.g. not knowing where to go for care or being unable to find a provider) may reflect a lack of awareness about the 9/11 treatment programs that can be addressed with individualized outreach programs. Research is needed to better understand the real and perceived barriers to 9/11-related health care, particularly among those with comorbid and more severe mental health symptoms who report the highest levels of UMHCN, so that these barriers can be removed and individuals can be connected to services.

A variety of broad public education campaigns have been conducted to reach 9/11-exposed individuals and inform them of treatment options. In addition, several outreach programs are in place to reach those eligible for 9/11-related treatment programs. For example, since 2009 the WTCHR has conducted the Treatment Referral Program (TRP), which contacts enrollees who lived or worked in the 9/11 disaster area and report a physical and/or mental health symptom to encourage them to seek care from 9/11-specialty providers [34]. Such programs attempt to reduce the cost and access barriers reported in this study by connecting individuals to mental health services at no out of pocket cost at a specific location. TRP staff trained in motivational interviewing engage with symptomatic enrollees to discuss their concerns about the decision to seek treatment in an attempt to address attitudinal barriers. To date, more than 1,100 enrollees have made their first appointment with a 9/11-specialty care site through the TRP.

### Strengths and limitations

The diversity and size of the Registry allows for reporting of levels of UMHCN across a range of sociodemographic characteristics and accounts for a large number of symptomatic individuals with reported exposure to the 9/11 attacks in NYC. In addition, the study was able to describe the level of UMHCN among those with mental health symptoms using two well-validated and widely used measures, the PHQ-8 and the PCL. A limitation is that UMHCN is measured with a single survey item that is subject to measurement error. Respondents likely evaluate UMHCN not only based on clinical need but varying expectations about health services they should receive, knowledge of the mental health system, and other factors [35]. For example, some may have interpreted the UMHCN question to include a limited set of mental health treatments rather than the full range of alternatives available to them. The nine options available to respondents to specify the reason for UMHCN represent a wide range of factors but may still not capture the full range of individual experience.

Registry enrollees that were self-identified may not be representative of those exposed. To minimize selection bias, the WTCHR was designed based on a comprehensive set of data sources including lists of likely exposed individuals to maximize coverage of the eligible population [14]. In this study, list-identified respondents were more likely to report UMHCN as compared to self-identified enrollees (OR = 1.1, 95% CI = 1.0, 1.3), but addition of a control for list-identification as compared to self-identification did not change the multivariate results shown substantially. Among the Wave 1 enrollees who were eligible, 63% participated in the Wave 3 Registry survey [24]. We were unable to ascertain differences in Wave 1 or Wave 2 UMHCN among Wave 3 participants and non-participants because the measure of UMHCN we analyzed in this study was not administered in the two earlier surveys. The association of Wave 3 survey participation status with probable PTSD at Wave 1 was statistically significant but small in magnitude. Enrollees who completed all 3 Registry surveys were less likely to have probable PTSD in the Wave 3 survey than those who completed Wave 3 but not Wave 2. Because we do not restrict this study to those who participated in all 3 Registry surveys we did not introduce bias due to the better health status of this group.

## Conclusion

This analysis informs the ongoing outreach efforts and research necessary to address the unmet mental health treatment need of individuals exposed to the 9/11 disaster. A significantly large group of individuals with PTSD or depression reported an UMHCN ten years after 9/11. Unmet need occurs due to a range of attitudinal, cost, and access barriers. Availability of health insurance and perceived social support served as protective factors against UMHCN due to access and cost barriers in particular. Individuals with severe conditions characterized by functional impairment and comorbid mental health symptoms are more likely to report UMHCN, with mental health service non-users displaying particularly strong attitudinal barriers to seeking treatment. Outreach programs are one avenue to address UMHCN and to encourage individuals to avail of affordable and accessible care from a specialty mental health provider available to those exposed to the 9/11 disaster.

## Competing interests

The authors declare that they have no competing interests.

## Authors’ contributions

SG, RB, and SS conceived the study with close input and supervision from MF and JC. SG performed the coding and statistical analysis of the data, and all authors participated in the interpretation of the findings. The manuscript was written by SG, with close input from RB and SS, and all authors participated in the revision of the manuscript for important intellectual content. All authors read and approved the final manuscript.

## Pre-publication history

The pre-publication history for this paper can be accessed here:

http://www.biomedcentral.com/1471-2458/14/491/prepub
